# Point-of-Care Diagnostic Tests for Detecting SARS-CoV-2 Antibodies: A Systematic Review and Meta-Analysis of Real-World Data

**DOI:** 10.3390/jcm9051515

**Published:** 2020-05-18

**Authors:** Matteo Riccò, Pietro Ferraro, Giovanni Gualerzi, Silvia Ranzieri, Brandon Michael Henry, Younes Ben Said, Natalia Valeryevna Pyatigorskaya, Elena Nevolina, Jianhong Wu, Nicola Luigi Bragazzi, Carlo Signorelli

**Affiliations:** 1 AUSL–IRCCS di Reggio Emilia, Servizio di Prevenzione e Sicurezza negli ambienti di Lavoro (SPSAL), I-42122 Reggio Emilia (RE), Italy; 2 ASL Foggia, Dipartimento di Prevenzione, Servizio Prevenzione e Sicurezza Ambienti Lavoro, Piazza Pavoncelli 11, I-41121 Foggia (FG), Italy; dott.pietro.ferraro@gmail.com; 3 Department of Medicine and Surgery, School of Medicine, University of Parma, 43123 Parma (PR), Italy; gualerzi@gmail.com; 4 Department of Medicine and Surgery, School of Occupational Medicine, University of Parma, I-43123 Parma (PR), Italy; silvia.ranzieri@studenti.unipr.it; 5 Cardiac Intensive Care Unit, The Heart Institute, Cincinnati Children’s Hospital Medical Center, 3333 Burnet Avenue, Cincinnati, OH 45229-3026, USA; brandon.henry@cchmc.org; 6 Department of Industrial Pharmacy, Sechenov First Moscow State Medical University (Sechenov University), 119991 Moscow, Russia; younisbensaid@gmail.com (Y.B.S.); osipova-mma@list.ru (N.V.P.); 7 Executive Director of the Union “National Pharmaceutical Chamber”, 125009 Moscow, Russia; rapalatainfo@gmail.com; 8 Laboratory for Industrial and Applied Mathematics (LIAM), Department of Mathematics and Statistics, York University, 4700 Keele Street, Toronto, ON M3J 1P3, Canada; wujh@yorku.ca; 9 School of Medicine, Vita-Salute San Raffaele University, 20132 Milan (MI), Italy; signorelli.carlo@hsr.it

**Keywords:** SARS-CoV-2, COVID-19, point-of-care diagnostics, rapid testing, systematic review and meta-analysis, real-world data

## Abstract

SARS-CoV-2 is responsible for a highly contagious infection, known as COVID-19. SARS-CoV-2 was discovered in late December 2019 and, since then, has become a global pandemic. Timely and accurate COVID-19 laboratory testing is an essential step in the management of the COVID-19 outbreak. To date, assays based on the reverse-transcription polymerase chain reaction (RT-PCR) in respiratory samples are the gold standard for COVID-19 diagnosis. Unfortunately, RT-PCR has several practical limitations. Consequently, alternative diagnostic methods are urgently required, both for alleviating the pressure on laboratories and healthcare facilities and for expanding testing capacity to enable large-scale screening and ensure a timely therapeutic intervention. To date, few studies have been conducted concerning the potential utilization of rapid testing for COVID-19, with some conflicting results. Therefore, the present systematic review and meta-analysis was undertaken to explore the feasibility of rapid diagnostic tests in the management of the COVID-19 outbreak. Based on ten studies, we computed a pooled sensitivity of 64.8% (95%CI 54.5–74.0), and specificity of 98.0% (95%CI 95.8–99.0), with high heterogeneity and risk of reporting bias. We can conclude that: (1) rapid diagnostic tests for COVID-19 are necessary, but should be adequately sensitive and specific; (2) few studies have been carried out to date; (3) the studies included are characterized by low numbers and low sample power, and (4) in light of these results, the use of available tests is currently questionable for clinical purposes and cannot substitute other more reliable molecular tests, such as assays based on RT-PCR.

## 1. Introduction

The “Severe Acute Respiratory Syndrome coronavirus type 2” (SARS-CoV-2) is an enveloped, single-stranded, positive-sense RNA virus, responsible for a highly contagious infection, known as “coronavirus disease 19” (COVID-19). SARS-CoV-2 was discovered in late December 2019 and, since then, has spread out from Wuhan, Hubei province, People’s Republic of China, into numerous countries worldwide, becoming a global pandemic [1,2,3].

Timely and accurate COVID-19 laboratory testing is an essential step in the management of the COVID-19 outbreak [4]. To date, both the “European Centre for Disease Prevention and Control” (ECDC) and the “World Health Organization” (WHO) have recommended the use of an assay based on the reverse-transcription polymerase chain reaction (RT-PCR) in respiratory samples as gold standard for the COVID-19 diagnosis [4,5,6]. Unfortunately, RT-PCR is affected by several practical limitations, including a relatively invasive sampling, a time-consuming procedure to process and generate results, the need for specialized operators and certified laboratories [7]. This makes the use of the RT-PCR particularly challenging in resource-limited settings. Moreover, a globally increased demand for diagnostic tests is limiting the availability of operational material for both respiratory sample collection and molecular diagnostics [5,7,8,9]. 

Consequently, alternative diagnostic methods are urgently required, for alleviating the pressure on laboratories and healthcare facilities, and in particular point-of-care rapid diagnostic tests (POCT) [4,7,8,10,11]. POCT are small stand-alone tests that are relatively simple to perform. Differently from other molecular tests, they can be deployed at the site of the triage, but also outside hospitals and other healthcare facilities. Furthermore, they do not require specialized, time-consuming training and can be, then, conducted also by low trained staff personnel, round-the-clock and on samples that do not need to be particularly processed. By definition, they provide test results in a short amount of time (from a few minutes to hours), enabling a swift patient management. Moreover, they can be employed by decentralized testing infrastructure, which makes them particularly attractive for the management and containment of outbreaks. Even though previous experiences with POCT targeting influenza antigens suffered from poor sensitivity [9], analogous tests for SARS-CoV nucleocapsid [12] as well as tests detecting viral RNA [13,14,15] are particularly promising, and may significantly improve our testing capacity to enable large-scale screening and ensure a timely therapeutic intervention. Still, among all POCT, assays able to detect antibody responses have become of significant interest, as such tests could not only allow a rapid diagnosis of acute infection, but could also complement nucleic acid testing by identifying subjects who have recovered from a previous infection, contributing to a better understanding of SARS-CoV-2 burden of disease [16,17].

Despite several stakeholders urging for their diffuse deployment [18], performance characteristics of rapid tests for SARS-CoV-2 antibodies remain largely uncertain [19]. Some conflicting results have been reported, suggesting the need for an updated synthesis of the literature to better inform health policies and guidelines. Therefore, the present systematic review and meta-analysis was undertaken to explore the reliability of rapid diagnostic tests for SARS-CoV-2 antibodies in the management of the COVID-19 outbreak.

## 2. Experimental Section

This systematic review and meta-analysis of the literature has been conducted following the “Preferred Reporting Items for Systematic Reviews and Meta-Analysis” (PRISMA) guidelines [20]. We searched different scholarly databases (namely, PubMed/MEDLINE and EMBASE) as well as pre-print servers including medrxiv.org for relevant studies from inception up to 13 April 2020, without applying any backward chronological restrictions. The search strategy was a combination of the following keywords (free text and Medical Subject Heading [MeSH] terms, where appropriate): (COVID-19 OR SARS-CoV-2 OR Coronarivus) AND (point-of-care OR rapid test OR fast test). 

Records were handled using references management software (Mendeley Desktop Version 1.19.5, Mendeley Ltd, London, UK, 2019), and duplicates were removed. Articles eligible for review were original research publications available online or through inter-library loan. A language filter was applied, by retaining articles written in Italian, English, German, French or Spanish, the languages spoken by the investigators. 

Only articles (a) dealing with commercially available point-of-care diagnostic tests for SARS-CoV-2 antibodies (IgM/IgG); (b) including COVID-19 cases diagnosed by means of RT-PCR; (c) reporting the raw number of true positive/true negative, and false positive/false negative results were eligible for the full review. Two independent reviewers (G.G. and S.R.) reviewed titles, abstracts, and the full text of articles. Titles were screened for relevance with respect to the subject under study. 

Any articles reporting original results, which met one or more of the inclusion criteria, were retained for the full-text review. The investigators independently read full-text versions of eligible articles. Disagreements were resolved by consensus between the two reviewers; when it was not possible to reach consensus, input from a third investigator (M.R.) was searched and obtained. Further studies were retrieved from reference lists of relevant articles and consultations with experts in the field. 

Data extracted included:

(1) Settings of the study;
(2) Characteristics of reference cases;
(3) Characteristics of reference diagnostic test;
(4) Characteristics of the commercially available point-of-care test employed; and,
(5) Total number of true positive, true negative, false positive, false negative cases.

We first performed a descriptive analysis to report the characteristics of the included studies. The accuracy of each study was measured as sensitivity (Se), specificity (Sp), positive and negative likelihood ratio (PLR and NLR), diagnostic odds ratio (DOR), accuracy and Cohen’s “kappa”. Sensitivity can be defined as the proportion of positive cases among people with a given disease, and specificity as the proportion of negative cases among people without that disease. 

The likelihood ratios express the likelihood that a given test result would be expected in a patient with a certain disorder compared to the likelihood of that same result among subjects without that disorder; PLR was calculated by dividing the pooled Se by 1–Sp; NLR was calculated by dividing 1–Se by Sp. The DOR expresses how much greater the odds of having the disorder are for the people with a positive test result than for the people with a negative test result. It is a single measure of diagnostic test performance that combines both likelihood ratios by dividing PLR by NLR. Cohen’s kappa coefficient is a statistical measure that is used to quantitatively assess inter-rater reliability (and also intra-rater reliability) for qualitative (i.e., categorical) items. In other words, it measures the agreement between two raters who each classify N items into C mutually exclusive categories. Cohen’s kappa values <0.600 suggest “*weak*” to “*none*” agreement, while values 0.600 to 0.799 suggest a “*moderate*” agreement, 0.800 to 0.900 a “*strong*” agreement, and >0.900 an “*almost perfect*” agreement.

Pooled Se, Sp, PLR, NLR, accuracy, Cohen’s kappa and DOR were calculated by combining each study’s results, using a random-effects model. For DOR, a correction factor of one half was added to each cell to avoid calculation problems by having a value of zero in the 2 × 2 table. 

Potential heterogeneity for threshold effect (i.e., differences in Se and Sp occurring because of different cut-offs used in different diagnostic kits) was assessed by reporting accuracy estimates from each study in a summary receiver operating characteristic (sROC) space and subsequent visual inspection of the resulting curves: i.e., a substantial difference in curves suggested a possible heterogeneity for threshold effect, while the overlap of curves was reported as a sign of its absence [21].

I^2^ statistics was then calculated to quantify the amount of inconsistency between included studies; it estimates the percentage of total variation across studies that is due to heterogeneity rather than to chance. I^2^ values ranging from 0% to 25% were considered to represent low heterogeneity, from 26% to 50% moderate heterogeneity and above 50% substantial heterogeneity, being pooled using a fixed-effects model because of the reduced number of samples eventually included. 

To investigate publication bias, contour-enhanced funnel plots were initially generated: publication bias was evaluated by testing the null hypothesis that publication bias does not exist by means of the regression test for funnel plot asymmetry. The null hypothesis was rejected if the *p*-value was less than 0.10. 

All calculations were performed in R (version 3.6.1; R Core Team, 2017. R: A language and environment for statistical computing. R Foundation for Statistical Computing, Vienna, Austria. URL https://www.R-project.org/) and RStudio (version 1.2.5019, RStudio PBC, 250 Northern Ave, Boston, MA, USA) software by means of *meta* (version 4.9-9) and *mada* (version 0.5-9) packages. Both packages are open-source add-ons for conducting meta-analyses.

## 3. Results

**Figure 1:**
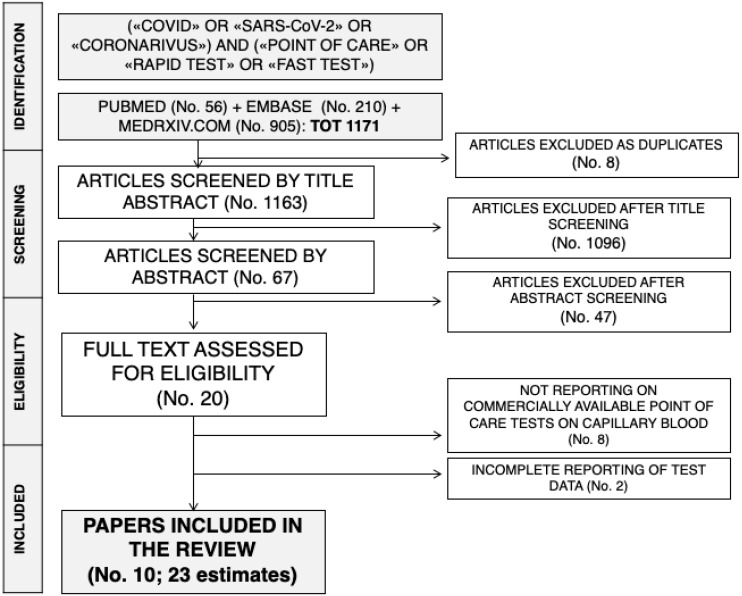
The process of studies retrieval and inclusion adopted in the present systematic review and meta-analysis. A total of 10 studies with 23 estimates were retrieved.

The initial literature search yielded a pool of 1166 items. After removing duplicates (n = 8), 1158 articles were screened by title and abstract. One thousand and ninety-six were removed after title screening and 62 were subsequently screened by abstract. Forty-seven were excluded based on abstract screening and, finally, 18 articles were assessed for eligibility, and reviewed by full-text. Seven studies did not report data on commercially available tests for SARS-CoV-2 antibodies, while two articles reported only incomplete data, and were then excluded. Ten studies with 24 estimates were eventually retrieved [7,8,10,11]. One of the studies reported a total of six estimates for six diagnostic kits [8]: as one of them included too few data to allow a proper analysis, the present systematic review and meta-analysis included a total of 23 estimates. The process of studies retrieval and inclusion is pictorially shown in Figure 1.

The main characteristics of the studies included in the present systematic review and meta-analysis are reported in Table 1. Briefly, eight of the included reports were preprints, with only the study of Cassaniti et al. [7] and Döhla et al. [22] having received a full peer-review process. Overall, we retrieved nine estimates from the study of Adams et al. [23], five estimates from the study of Lassauniére et al. [8], two estimates from the aforementioned report of Cassaniti et al. [7], while all other studies include a single estimate each [10,11,24,25,26,27]. A total of nine diagnostics point-of-care tests were assessed (i.e., Dynamiker; CTK Biotek, AutoBio Diagnostics, Artron Laboratories, Acro biotech, VivaDiag, AllTest Biotech, Premier Biotech, Zhuhai Livzon Diagnostics), with further studies assessing POCT by undisclosed manufacturers [22,23,26]. 

More specifically, three estimates were obtained through the VivaDiag kit [7,10], two estimates by means of Artron diagnostic tests [8,27], while other known manufacturers were assessed by a single estimate each [8,11,24,25]. Overall, our meta-analysis included a total of 2252 samples (1030 SARS-CoV-2 positive samples vs. 1222 SARS-CoV-2 negative samples): the sample size for every single estimate ranged from 20 to 212. The majority of samples (54.1%) were assessed by kits from manufacturers whose identity was undisclosed by researchers. Regarding the remaining estimates, the VivaDiag test was assessed on a total of 300 samples (13.3%), followed by Artron Laboratories diagnostic kit (234 samples, 10.4%), and the rapid tests from Zhuhai Livzon Diagnostics (126 samples, 5.6%), AllTest Biotech kit (100 samples, 4.4%), Dynamiker CTK Biotech, Autobio (each one 62 samples, 2.8%), and Acro Biotech (20 samples, 2.2%).

**Figure 2:**
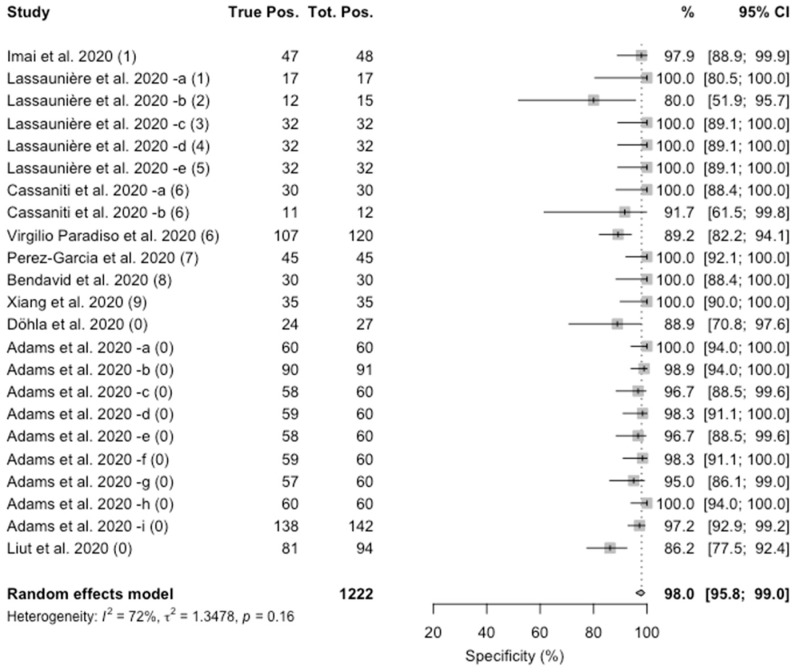
Forest plot representing the estimated pooled specificity of studies on Point-of-Care rapid tests for IgM/IgG antibodies for SARS-CoV-2. Mean specificity is 98.0% (95%CI 95.8–99.0). Notes on manufacturers of assessed kits: (1) Artron laboratories; (2) Acro Biotech; (3) AutoBio Diagnostics; (4) Dynamiker; (5) CTK Biotech; (6) VivaDiag; (7) AllTest Biotech; (8) Premier biotech; (9) Zhuhai Livzon Diagnostics; (0) undisclosed manufacturer.

Concerning specificity, the value ranged from 80% to 100%. The pooled estimate was 98.0% (95%CI 95.8–99.0), with a substantial heterogeneity (I^2^ = 72%, *p* = 0.16), as shown in Figure 2. 

**Figure 3:**
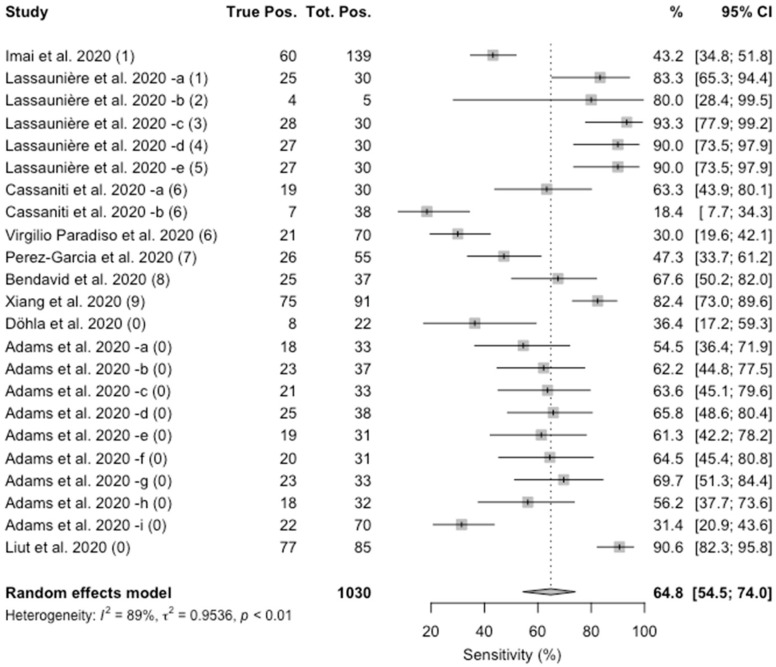
Forest plot representing the estimated pooled sensitivity of studies on Point-of-Care rapid tests for COVID-19. Mean specificity is 64.8% (95%CI 54.5–74.0). Notes on manufacturers of assessed kits: (1) Artron laboratories; (2) Acro Biotech; (3) AutoBio Diagnostics; (4) Dynamiker; (5) CTK Biotech; (6) VivaDiag; (7) AllTest Biotech; (8) Premier biotech; (9) Zhuhai Livzon Diagnostics; (0) undisclosed manufacturer.

As shown in Figure 3, the value of specificity ranged from 18.4% to 93.3%. The pooled estimate was 64.8% (95%CI 54.5–74.0), and again a substantial heterogeneity was identified (I^2^ = 86%; *p* < 0.01).

Contour-enhanced funnel plots (See Appendix A), showed evidence of publication bias for both specificity (t = 2.0987; df = 21, *p* = 0.0005) and sensitivity (t = 2.7869; df = 21, *p* = 0.0111). 

**Figure 4:**
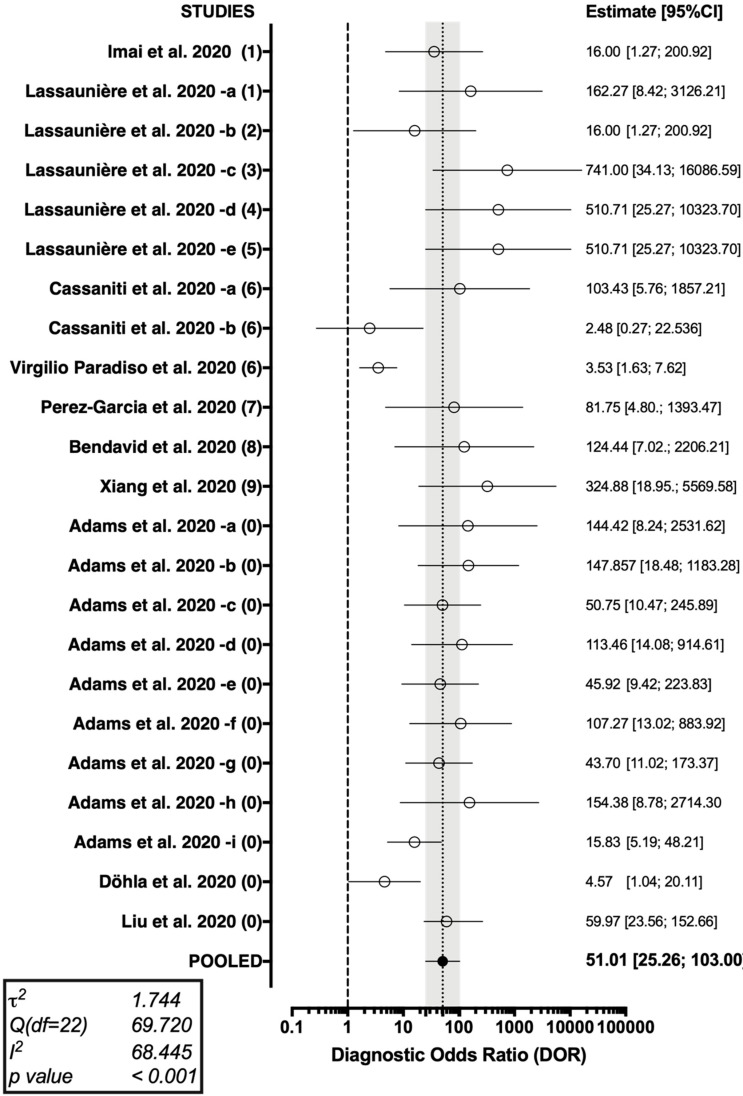
Forest plot representing the pooled diagnostic odds ratio of the studies on Point-of-Care COVID-19 diagnostics. A substantial heterogeneity in reported studies was identified (I^2^ 68.45%, Cochran’s Q: 69.720 (22 df, *p* < 0.001)).

The forest plot representing the pooled DOR (51.01 [95%CI 25.26–103.00]) from the studies on Point-of-Case diagnostic test for SARS-CoV-2 antibodies is shown in Figure 4. 

As shown in Table 2, a cumulative PLR of 14.2 (95%CI 8.7; 23.2) was determined, with a correspondent NLR of 0.4 (95%CI 0.2; 0.5), i.e., while a positive test was associated with a relatively strong evidence of disease, a negative one was associated with a residual chance of being actually affected by SARS-CoV-2 infection. In effect, Cohen’s kappa was estimated at 0.594 (95%CI 0.496; 0.691), suggesting that the agreement between rapid tests based on the assessment of serum antibodies and RT-PCR may be assumed to be weak.

In order to exclude that the differences in overall performance may have been associated with a different threshold level among studies, sROC of two distinctive models were determined, either using the overall DOR by combining each study’s diagnostic odds ratio (random-effects model (Appendix B), AUC 0.934), or a fixed (unweighted) method (AUC 0.933). The slight difference between the symmetric and asymmetric sROC curves indicates that a null to weak threshold effect is present.

## 4. Discussion

SARS-CoV-2 is a highly contagious and rapidly evolving infectious agent. Even though a significant share of SARS-CoV-2 positive patients evolves to a severe or even critical disease, needing mechanical ventilation, sub-intensive or even intensive care [1,28,29,30], COVID-19 has a substantially favorable clinical course in nearly 80% of infected patients, being most of them asymptomatic or displaying only mild and totally nonspecific symptoms [1,30]. As a consequence, POCT would have not only the potential to significantly improve current testing capacity for COVID-19, giving results in a quick time with a low-cost detection, avoid overwhelming and straining limited healthcare resources and help overcome organizational issues in low-resource contexts [31], but may also guarantee a better understanding of SARS-CoV-2 burden of disease, pointing to more appropriate public health interventions, such as case definition, contact tracing and patient isolation [4,7,8,9,10,11].

However, by collecting current evidence on commercially available POCT for SARS-CoV-2 antibodies, our review stresses that these kits may be scarcely reliable in real-world settings. First, when comparing our results with the gold-standard (i.e., RT-PCR), we estimated a pooled Cohen’s kappa of 0.594 (95%CI 0.496–0.691), meaning that only a weak agreement between POCT and more conventional (but also time consuming and somewhat invasive) nasal swabs for RT-PCR may be assumed. Moreover, we reported a pooled sensitivity of only 64.8%. In other words, the resulting rate of false negative cases may be substantial; not coincidentally, both WHO and ECDC, but also some national stakeholders, have pointed out that the use of these tests should be restrained to the research settings only, avoiding their use in any other setting, even for clinical decision-making [4,18,19]. Interestingly, while some authors initially reported some concerns towards the possible cross-reactivity of SARS-CoV-2 antigens with other viral pathogens, the large majority of samples reported high or even very high specificity, with the worse performance from an assessment including very few samples (No. of total samples = 20) [8]. Still, a more appropriate analysis of our results suggests that reliability of POCT for viral antibodies may be even more feeble. In fact, both specificity and sensitivity of assessed tests were affected by substantial heterogeneity (72% and 89%, respectively), and high risk for reporting bias, particularly for specificity assessment, i.e., a seemingly deliberate exclusion from the available literature of tests affected by lower specificity.

Some explanations for such unsatisfying performances may be drawn from our current understanding of SARS-CoV-2 infection. As previously suggested, the median time of antibody appearance ranges between 3–6 days after the onset of symptoms for both IgM/IgA. Even though within 19 days from the onset of symptoms nearly all patients test positive for IgG, their onset may be particularly delayed, up to 18 days from the earlier symptoms [17,31]. As a consequence, it is reasonable that in some of the studies included in our analysis, a POCT performed too early may have failed to properly characterize the status of the patients not only because of intrinsic detection limits, but also because of a late surge of IgM/IgG. In fact, as pointed out in some studies [8,23,27,32], sensitivity of available POCT tests for SARS-CoV-2 increases after the first two weeks from the beginning of the symptoms. Therefore, we could speculate that our eventual estimates may have forcefully underestimated the potential sensitivity of available kits. However, in a real-world perspective, such limits of POCT for SARS-CoV-2 are particularly frustrating. As the large majority of SARS-CoV-2 cases are actually asymptomatic, and even among milder cases most of symptoms may be unnoticed, particularly when referring to their onset (i.e., mild conjunctivitis, particularly in spring season; cough; fever, etc.), patients may be extensively tested in earlier stages of the SARS-CoV-2 infection, with the aim to identify subjects potentially benefiting from prompt and proper supportive therapy. Unfortunately, our results suggest that antibody-based POCT may be inappropriate for such a purpose. In such settings, POCT based on viral RNA or antigens may be potentially more cost-effective [12,13,14,15]. On the contrary, the analysis of sROC curves, their analogies, and the substantial correspondence of the AUCs from fixed- and random-effects models, hint towards a possible irrelevance of the actual serum levels for IgM/IgG, i.e., it is reasonable that subjects who developed a proper but somewhat scarce immune response to the virus, as well as patients in the very late phases of the infection (i.e., viral clearance) do not have an increased risk to be improperly diagnosed as negative when compared to patients in the proper “diagnostic open window”.

Despite the increasing concerns towards the limited reliability of POCT for SARS-COV-2 antibodies, in several Western countries there is an increasing public call to the use of point-of-care diagnostic tests as a sort of post-lockdown mass screening in order to allow a prompt re-admission of “healthy” workers (either naïve or naturally immunized against SARS-CoV-2), guaranteeing a sort of “immunity passport”. Even though a recent report has apparently guaranteed that potentially neutralizing IgG levels may last much longer than previously suspected (i.e., based on our understanding of other Coronavirus) [17], the extensive referral to IgG/IgM values in settings other than the epidemiologic one may be incautious [16,19]. In fact, an inappropriate trust in POCT with a very low sensitivity may lead to misdiagnosing true positive but asymptomatic cases, potentially eliciting new outbreaks in the very same settings that such tests should contribute to protect (e.g., retirement homes, healthcare settings, workplaces, etc.) [16,19,23].

Notwithstanding the relative importance of our results, some significant limitations should be stressed. First and foremost, a significant share of included studies were retrieved from a pre-print platform (i.e., medrxiv.org) [8,10,11,23,24,25,26,27], while only two reports, to date, have been properly received a peer-review process [7,22]. Second, our meta-analysis did not take in account the presumptive delay between test performing and the reported onset of the symptoms, as not regularly available from index studies. As a consequence, it is possible that most of commercial kits that did not perform particularly well may have been impaired from the source, being employed in an inappropriate timeframe [7,8,10,11,31], underestimating their actual sensitivity in optimal settings. As a consequence, we suggest that our results should be retained cautiously and not as a comparative assessment of commercially available kits. Third, the studies included are generally characterized by low numbers and low sample power; particularly in one study [8], two commercial kits were assessed with very few cases, forcing us to exclude one estimate from the analyses.

In conclusion, the use of available POCT for SARS-CoV-2 antibodies is currently questionable for clinical purposes and cannot substitute other more reliable molecular tests, such as assays based on the RT-PCR. However, given the limitations of the present review, such as the small number of studies, the small sample sizes and the high, statistically significant amount of heterogeneity among studies, further high-quality research in the field is warranted. 

## Figures and Tables

**Table 1:** Summary of studies included in the meta-analysis (Note: TP = No. of True Positive Cases; FP = No. of False Positive Cases; FN = No. of False Negative Cases; TN = No. of True Negative Cases; Se. = sensitivity; Sp. = specificity; PPV = predicted positive value; PNV = predicted negative value; Cohen’s Kappa values should be interpreted as follows: 0.0–0.20 no agreement, 0.21–0.39 minimal agreement, 0.40–0.59 weak agreement, 0.60–0.79 moderate agreement, 0.80–0.90 strong agreement, > 0.90 almost perfect agreement; POCT = Point-of-Care Test).

Reference	Characteristics of the Samples	Commercial Test	No. of Samples	TP	FP	FN	TN	Se.	Sp.	PPV	PNV	Accuracy	Cohen’s Kappa
Imai et al. 2020 [27]	139 samples from 112 COVID-19 patients, 48 negative patients from Saitama Hospital	Artron laboratories	187	60	1	79	47	42.2%	97.9%	98.4%	37.3%	57.2%	0.268
Lassaunière et al. 2020 [8]	30 COVID-19 confirmed cases admitted to intensive care unit vs. 10 samples from healthy donors + patients with previous history of coronarivus infection (N = 5), non-coronavirus respiratory infection (N = 45), dengue (N = 9), CMV (N = 2), EBV (N = 10).	Artron Laboratories	47	25	0	5	17	83.3%	100%	100%	77.3%	89.4%	0.783
		Acro Biotech	20	4	3	1	12	80.0%	80.0%	57.1%	92.3%	80.0%	0.529
		AutoBio Diagnostics	62	28	0	2	32	93.3%	100%	100%	94.1%	96.8%	0.935
		Dynamiker	62	27	0	3	32	90.0%	100%	100%	91.4%	95.2%	0.903
		CTK Biotech	62	27	0	3	32	90.0%	100%	100%	91.4%	95.2%	0.903
Cassaniti et al. 2020 [7]	30 negative healthy controls vs. 30 COVID-19 cases tested with both POCT and RT-PCR	VivaDiag	60	19	0	11	30	63.3%	100%	100%	73.2%	81.7%	0.633
	50 subjects with suspected COVID-19 sequentially tested with POCT and RT-PCR	VivaDiag	50	7	1	31	11	18.4%	91.7%	87.5%	26.2%	36.0%	0.054
Virgilio Paradiso et al. 2020 [10]	191 cases with suspected COVID-19 sequentially tested with POCT and RT-PCR	VivaDiag	191	21	13	49	107	30.0%	89.2%	61.8%	68.6%	67.4%	0.215
Perez-Garcia et al. 2020 [11]	45 healthy controls vs. 55 SARS-CoV-2 cases	AllTest Biotech	100	26	0	29	45	47.3%	100%	100%	60.8%	71.0%	0.447
Döhla et al. 2020 [22]	39 cases randomly selected among subjects referring to a German COVID-19 screening center	Undisclosed manufacturer	49	8	3	14	27	36.4%	88.9%	72.7%	63.2%	65.3%	0.265
Bendavid et al. 2020 [24]	30 orthopedic patients, samples collected before COVID-19 pandemics; 37 PCR confirmed COVID-19 samples	Premier Biotech	67	25	0	12	30	67.6%	100%	100%	71.4%	82.1%	0.651
Xiang et al. 2020 [25]	35 healthy individuals; 91 RT-PCR confirmed plasma samples from COVID-19 patients	Zhuhai Livzon Diagnostics	126	75	0	16	35	82.4%	100%	100%	68.6%	87.3%	0.723
Adams et al. 2020 [23]	Random samples from a pool of 40 SARS-CoV-2 positive blood samples, and 50 SARS-CoV-2 negative blood samples	Undisclosed manufacturers	93	18	0	15	60	54.5%	100%	100%	80.0%	54.5%	0.608
			128	23	1	14	90	62.2%	98.9%	95.8%	86.5%	62.2%	0.682
			93	21	2	12	58	63.6%	96.7%	91.3%	82.0%	63.6%	0.647
			98	25	1	13	59	65.8%	98.3%	96.2%	81.9%	65.8%	0.681
			91	19	2	12	58	61.3%	96.7%	90.5%	82.9%	61.3%	0.629
			91	20	1	11	59	64.5%	98.3%	95.2%	84.3%	64.5%	0.682
			93	23	3	10	57	69.7%	95.0%	88.5%	85.1%	69.7%	0.679
			92	18	0	14	60	56.3%	100%	100%	81.1%	56.3%	0.626
			212	22	4	48	138	31.4%	97.2%	84.6%	74.2%	31.4%	0.340
Liu et al. 2020 [26]	179 consecutive patients, grouped by results of RT-PCR in SARS-CoV-2 positive (No.90), and negative ones (No.89)	Undisclosed manufacturer	179	77	13	8	81	90.6%	86.2%	85.6%	91.0%	88.3%	0.765

**Table 2:** Summary of the properties of point-of-care rapid diagnostic testing for the COVID-19.

Property	Value	95%CI
Sensitivity (%)	64.8	54.5; 74.0
Specificity (%)	98.0	95.8; 99.0
PLR	14.2	8.7; 23.2
NLR	0.4	0.2; 0.5
DOR	51.01	25.26; 103.00
Cohen’s kappa	0.594	0.496; 0.691

Abbreviations: DOR (Diagnostics Odds-Ratio); NLR (Negative Likelihood Ratio); PLR (Positive Likelihood Ratio).

**Figure A1:**
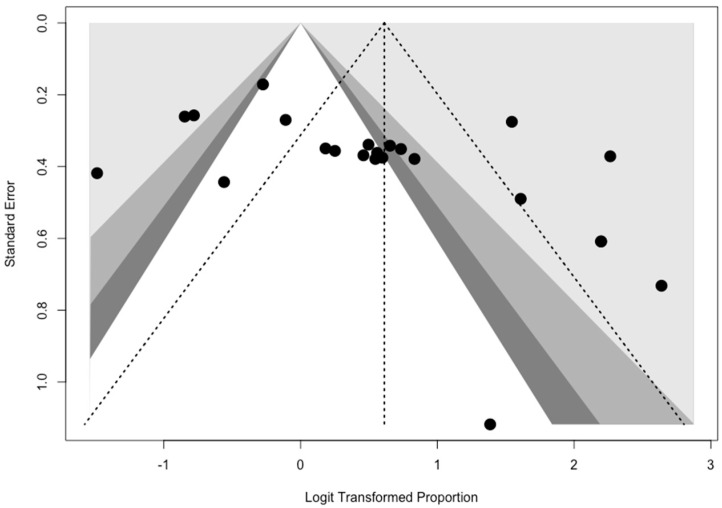
Funnel plot for sensitivity of studies included in the meta-analysis. Visual inspection suggested a significant asymmetry, with subsequent reporting bias.

**Figure A2:**
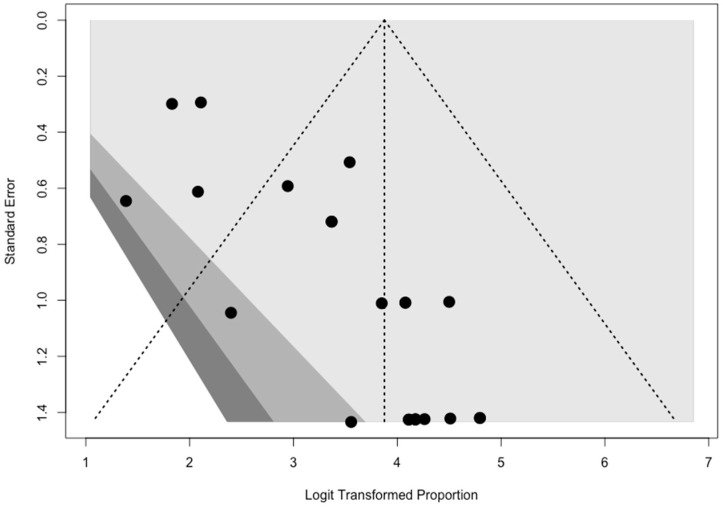
Funnel plot for specificity of studies included in the meta-analysis. Visual inspection suggested a significant asymmetry, with subsequent reporting bias.

**Figure A3:**
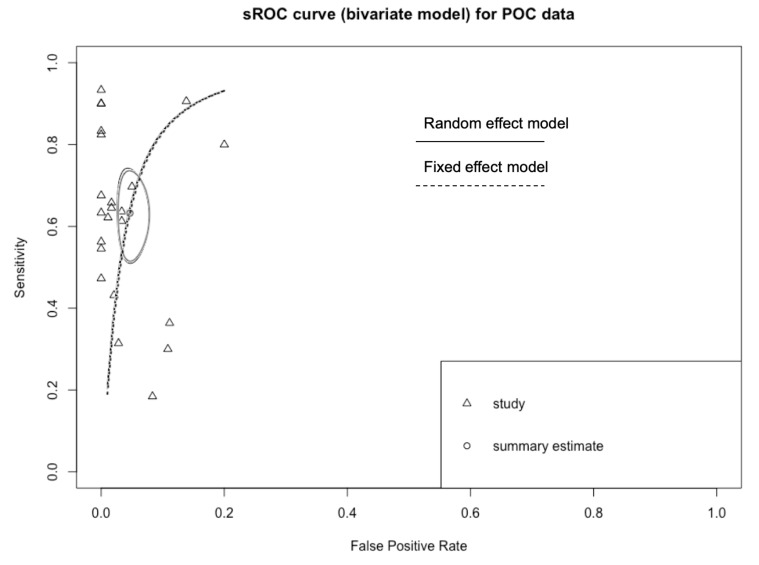
sROC curve for POC data. The slight differences between estimates from a random-effects model and a fixed-effects model suggest the absence of a threshold effect in diagnostic performances of assessed tests.
